# The value of GATA6 immunohistochemistry and computer-assisted diagnosis to predict clinical outcome in advanced pancreatic cancer

**DOI:** 10.1038/s41598-021-94544-3

**Published:** 2021-07-22

**Authors:** Kai Duan, Gun-Ho Jang, Robert C. Grant, Julie M. Wilson, Faiyaz Notta, Grainne M. O’Kane, Jennifer J. Knox, Steven Gallinger, Sandra Fischer

**Affiliations:** 1 Laboratory Medicine Program, University Health Network, 200 Elizabeth Street, Toronto, ON M5G 2C4 Canada; 2 Department of Laboratory Medicine and Pathobiology, University of Toronto, Toronto, ON Canada; 3 Ontario Institute for Cancer Research, Toronto, ON Canada; 4 Wallace McCain Centre for Pancreatic Cancer, Princess Margaret Cancer Centre, University Health Network, Toronto, ON Canada; 5 Division of Research, Princess Margaret Cancer Centre, University Health Network, Toronto, ON Canada; 6 Lunenfeld Tanenbaum Research Institute, Mount Sinai Hospital, Toronto, ON Canada; 7 Hepatobiliary/Pancreatic Surgical Oncology Program, University Health Network, Toronto, ON Canada; 8 Laboratory Medicine Program, University Health Network, 200 Elizabeth Street, Toronto, ON M5G 2C4 Canada

**Keywords:** Biomarkers, Pancreatic cancer

## Abstract

Combination chemotherapy, either modified FOLFIRINOX (mFFX) or gemcitabine–nabpaclitaxel, are used in the treatment of most patients with advanced pancreatic ductal adenocarcinoma (PDAC), yet robust biomarkers of outcome are currently lacking to guide regimen selection. Here, we tested GATA6 immunohistochemistry (IHC) as a putative biomarker in advanced PDAC. GATA6 is a transcription factor in normal pancreas development. Two pathologists, blinded to clinical and molecular data, independently assessed GATA6 IHC in biopsy specimens of 130 patients with advanced PDAC, in 2 distinct phases (without and with computer assistance using the open source software QuPath). Low GATA6 IHC expression was associated with shorter overall survival [median OS 6.2 months for patients with GATA6 low tumors vs. 11.5 months for patients with GATA6 high tumors, HR 1.66 (95% CI 1.15–2.40), *P* = 0.007]. Progression appears to be higher in GATA6-low tumors compared to GATA6-high tumors in patients treated with mFFX (*P* = 0.024) but not in patients treated with gemcitabine regimens. GATA6 IHC expression was significantly associated with molecular subtypes (*P* = 0.0003). Digital assistance markedly improved interrater concordance (Cohen’s kappa scores of 0.32 vs. 0.95). Our results provide strong evidence that GATA6 IHC can be used as a single biomarker in the clinic to predict clinical outcome in advanced PDAC, warranting further investigation in prospective clinical trials. These results provide the basis for an improved classification of PDAC and future biomarker design using digital pathology workflow.

## Introduction

Pancreatic ductal adenocarcinoma (PDAC) is expected to become the second leading cause of cancer-related deaths in North America by 2030^1^. Most patients present with advanced disease where the mainstay of treatment is combination chemotherapy [modified FOLFIRINOX (mFFX) or gemcitabine–nabpaclitaxel (GnP)] and outcomes remain poor with median survival less than 1 year^2–4^. Gene expression profiling in resected PDAC identified distinctive subtypes that do not yet impact clinical practice^5–7^. These include the 3-subtype (classical, quasimesenchymal, and exocrine-like) classification by Collisson et al., the 4-subtype (immunogenic, progenitor, ADEX, and squamous) classification by Bailey et al., and the 2-subtype (classical and basal-like) classification by Moffitt et al. Interestingly, the quasimesenchymal (Collisson), squamous (Baily) and basal-like (Moffitt) tumors align well across the classifiers and are all associated with poor prognosis. However, as these studies pertained mainly to resected PDAC, the translation of these findings to advanced stage PDAC remains unclear.

Recently, our group demonstrated that the Moffitt RNA signature can be modified to predict prognosis and chemotherapy response in patients with advanced stage PDAC^2,8^. We also showed that the expression of GATA6, a transcription factor required for normal pancreas development^9^, measured by RNA sequencing or in situ hybridization (ISH), could be used as a single surrogate biomarker to distinguish classical PDAC from the more chemoresistant basal-like PDAC, which is associated with poor prognosis^2,8,9^. However, the clinical applicability of these sequencing and hybridization modalities remain limited by technical cost, accessibility, and reporting time.

GATA6 immunohistochemistry (IHC) is therefore an attractive alternative assay since it is less costly, more widely available and can be readily integrated in the clinic by pathologists on routine FFPE tissue at the time of diagnosis. However, the clinical utility of GATA6 IHC as a biomarker remains unknown in advanced PDAC. Based on preliminary experience^2^, we found IHC to be less accurate than RNA-seq or ISH, possibly due to the subjective nature of visual assessment of IHC staining intensity. Recently, advances in digital pathology and image analysis have raised the possibility for computer assistance to improve evaluation of IHC biomarkers^10–12^. Here, we evaluated the utility of GATA6 IHC as a single putative biomarker in advanced PDAC and interrogated the value of computer assistance to improve its assessment.

## Material and methods

### Patient population

Advanced PDAC cases were obtained from the COMPASS trial, a prospective multi-institutional Canadian cohort study. Patient eligibility for the study has been described previously^2,8^. Briefly, patients required a radiologic or histologic diagnosis of locally advanced or metastatic PDAC, suitable for combination chemotherapy, and must consent to a fresh tumor biopsy prior to treatment start. Tissue samples consisted of needle-core biopsies taken from the primary lesion or any metastatic sites. Patients must not have had prior treatment for advanced disease. Treatment decisions were at the discretion of their medical oncologist and response to therapy was assessed using CT and measured using RECIST 1.1. Treatment details, including subsequent treatments, were prospectively collected using an electronic MEDIDATA database. The COMPASS trial has been approved by participating site Institutional Review Board (University Health Network, Toronto, Ontario, Canada; MUHC Centre for Applied Ethics, Montreal, Quebec, Canada; and Queen's University Health Sciences and Affiliated Teaching Hospitals Research Ethics Board, Kingston, Ontario, Canada); each patient provided written informed consent prior to study entry, and this study was performed in accordance with relevant guidelines and regulations.

### Reference molecular classification and GATA6 RNA expression

The reference molecular classification for each case was established through RNA-sequencing (RNA-seq) analysis performed at the Ontario Institute of Cancer Research (Ontario, Canada), as described previously^2,13^. Briefly, reads were aligned to the human reference genome (hg38) and transcriptome (Ensembl v84) using STAR v.2.5.2a (11). Duplicated reads were marked using Picard v. 1.121 (https://github.com/broadinstitute/picard). Gene expression was calculated in fragments per kilobase of exon per million reads mapped using the cufflinks package v. 2.2.1^14^. A modified Moffitt classification (classical vs basal-like) was also applied to each sample with sufficient RNA for analysis, as described previously^2,13^. GATA6 RNA expression levels was established using RNA-seq, as described previously^2,8^.

### GATA6 immunohistochemistry

GATA6 IHC was performed on formalin-fixed paraffin embedded (FFPE) biopsy specimen tissue of advanced PDAC as previously reported^2^. Briefly, we optimized a protocol for GATA6 IHC using a polyclonal anti-GATA6 antibody from R&D (catalog number AF1700), and secondary antibody from Vector (catalog number VECTABA5000). DAB + (3,3-diaminobenzidine tetrahydrochloride plus, DAKO, catalog number K3468) was used as chromogen and nuclei were counterstained with Mayer hematoxylin.

### Slide scanning and digital assistant development

GATA6 IHC slides of advanced PDAC were scanned with a Leica Aperio AT2 Scanner (Leica Biosystems, Vista, CA, USA) at 40 × magnification. Resulting whole-slide images (WSI) were imported into an open source software (QuPath v0.2.0) for pathologist viewing and assessment. A digital assistance tool was developed using the QuPath’s positive cell detection algorithm^15^. Detection parameters were customized and confirmed by a senior pathologist (S.E. Fischer) experienced in quantifying GATA6 IHC expression in PDAC.

### Pathologist assessment without and with digital assistance

**Figure 1:**
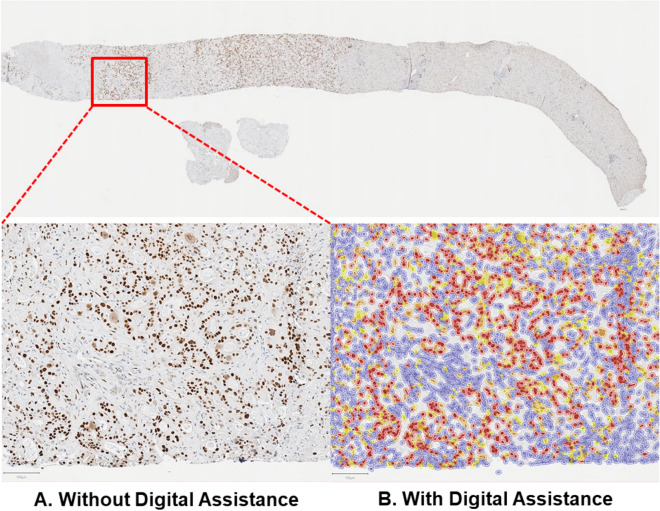
Pathologist workflow without and with digital assistance. In the assisted mode, the digital assistant provided GATA6 IHC expression for individual cells as a direct overlay. The pathologist had the option to toggle the digital assistant “off” (**A**) versus “on” (**B**) to better visualize tumor cells for assessment of GATA6 IHC expression. In both study phases (unassisted vs. assisted), pathologists recorded the final GATA6 IHC expression scores (0–4) for each case.

**Figure 2:**
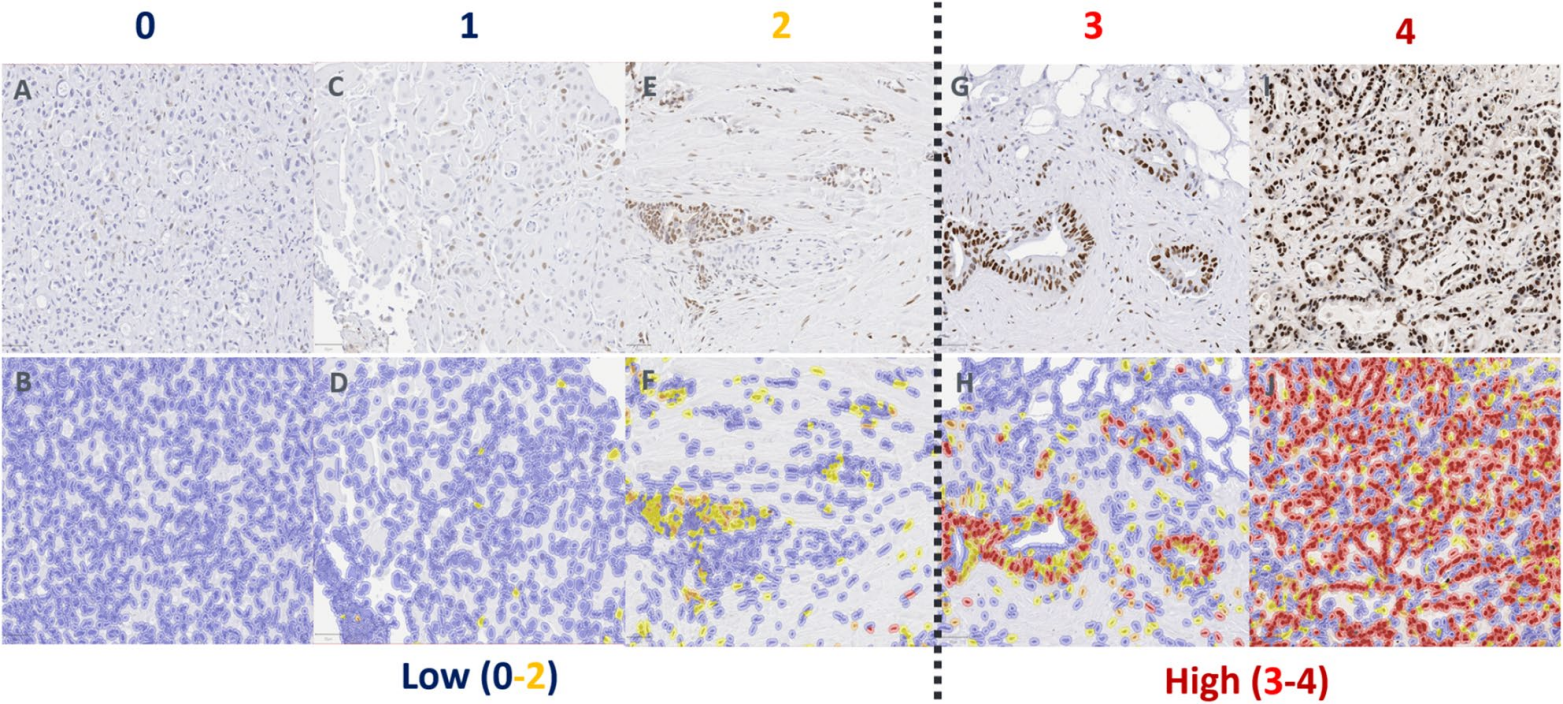
GATA6 IHC expression levels. No or weak GATA6 IHC expression (scores 0–1) without digital assistance (**A**,**C**) and with digital assistance (**B**,**D**) showing tumor as predominantly blue. Moderate GATA6 IHC expression (score 2) without digital assistance (**E**) and with digital assistance (**F**) showing tumor as predominantly yellow. (**D**) Strong GATA6 IHC expression (score 3) without digital assistance (**G**) and with digital assistance (**H**) showing tumor as predominantly red (≥ 50% of tumor cells). (**E**) Very strong GATA6 IHC expression (score 4) without digital assistance (**I**) and with digital assistance (**J**) showing tumor as nearly all red (≥ 90% of tumor cells). GATA6 IHC expression levels were regrouped into low (0–2) and high (3–4) groups to assess for an association with clinical outcome.

To compare prediction quality without and with digital assistance, 2 pathologists (P1 and P2), blinded to molecular and clinical data, independently assessed GATA6 IHC in biopsy specimens of 130 patients with advanced PDAC, in 2 distinct phases (without and with digital assistance). There was at least 4 weeks of washout period between the 2 phases, per consensus recommendations from the College of American Pathologists for avoiding short-term memory bias in digital pathologic validation studies^16^. In the assisted mode, the digital assistant provided GATA6 IHC semiquantitative (SQ) scores (0–4) for individual cells. The pathologist had the option to toggle on/off the GATA6 score indicator to better visualize tumor cells for assessment (Fig. 1). In both study phases, pathologists recorded the final GATA6 IHC SQ scores (0–4; Fig. 2) for each PDAC case.

### Statistical analysis

The performance metrics of individual pathologists, without and with digital assistance, were computed to assess for association with clinical outcome and known PDAC molecular subtypes (modified Moffitt, Collisson et al., Bailey et al.). Qualitative variables were compared by Fisher exact test, and quantitative variables by Wilcoxon rank-sum test for pairwise comparison and the Kruskal–Wallis test for multiple group comparison. All patients receiving at least one cycle of chemotherapy were included in the analysis of overall response rate (ORR). Survival curves were plotted using the Kaplan–Meier method and HRs were calculated using Cox proportional hazard regressions with *P* values calculated using the Wald statistic. All tests were two-sided. Multiple tests *P* values were adjusted using Benjamini and Hochberg method. Statistical significance was set at *P* = 0.05. Overall survival (OS) was assessed using Kaplan–Meier curves and compared between GATA6 IHC levels. After an initial analysis of all five levels, GATA6 IHC expression levels were regrouped into low (0–2) and high (3–4) groups to assess for an association with overall survival (OS) and overall response rate (ORR). The Cohen kappa was used to determine interrater reliability for class concordance. All analyses were conducted in R version 4.0.

## Results

### Patient characteristics

The clinical and pathologic characteristics of patients with advanced PDAC in our cohort are summarised in Table 1. One hundred and thirty patients with advanced PDAC from the COMPASS trial were included: 17, 24, 16, 63, and 10 patients had GATA IHC expression levels of 0, 1, 2, 3, and 4, respectively. After an initial analysis of all five levels, GATA6 IHC expression levels were regrouped into low (0–2) and high (3–4) groups. Using this classifier, 57 (44%) baseline tumor samples were GATA6-low and 73 (56%) GATA6-high. Baseline characteristics were similar between the groups (Table 1).

**Table 1:** Patient characteristics according to GATA6 IHC expression (n = 130).

	GATA6 Low (*N* = 57) *N* (%)	GATA6 High (*N* = 73) N (%)	*P*
Median age (years)	63.0 (42–81)	65.0 (29–77)	0.57
**Sex**			
Female	20 (35)	34 (47)	
Male	37 (65)	39 (53)	0.21
**Stage**			
Locally advanced	3 (5)	7 (10)	
Metastatic	54 (95)	66 (90)	0.51
**Race**			
White	38 (67)	57 (78)	
Asian	14 (25)	10 (14)	
African/other	1 (2)	5 (7)	
Unknown	4 (7)	1 (1)	0.15
**Prior resection**			
Yes	5 (9)	6 (8)	
No	52 (91)	67 (92)	1.00
**HRD genotype**			
Yes	5 (9)	6 (8)	
No	52 (91)	67 (92)	1.00
**First chemotherapy**			
mFFX	25 (44)	38 (52)	
GnP	24 (42)	33 (45)	0.85
None	8 (14)	2 (3)	

*GnP* gemcitabine–nabpaclitaxel, *mFFX* modified FOLFIRINOX.

### Response to chemotherapy according to GATA6 IHC expression

Of the 130 patients, 10 (7.7%) did not receive any chemotherapy and were considered nonevaluable (NE) and a further 10 patients (8%) died as a result of rapid functional decline prior to their first scan; 13 of these 20 had GATA6-low PDAC. Accordingly, RECIST response data were available for 110 patients (85%) including 44 patients with GATA6-low tumors and 66 with GATA6-high tumors. The ORR in GATA6-high PDAC was 41% versus 25% in GATA6-low PDAC (*P* = 0.104). In patients treated with mFFX and evaluable for response (n = 57), progression was evident in 39% of GATA6-low versus 12% of GATA6-high PDAC (*P* = 0.024). The ORR was 53% versus 26% in GATA6-high versus GATA6-low PDAC, respectively (*P* = 0.058). The numbers treated with GnP and available for response were smaller (n = 53), progression of disease was seen in 7 of 21 (33%) patients with GATA6-low versus 5 of 32 (16%) with GATA6-high tumors (*P* = 0.183). The ORR was 28% versus 24% in GATA6-high versus GATA6-low PDAC, respectively (*P* = 1.000).

### GATA6 IHC expression is associated with overall survival

**Figure 3:**
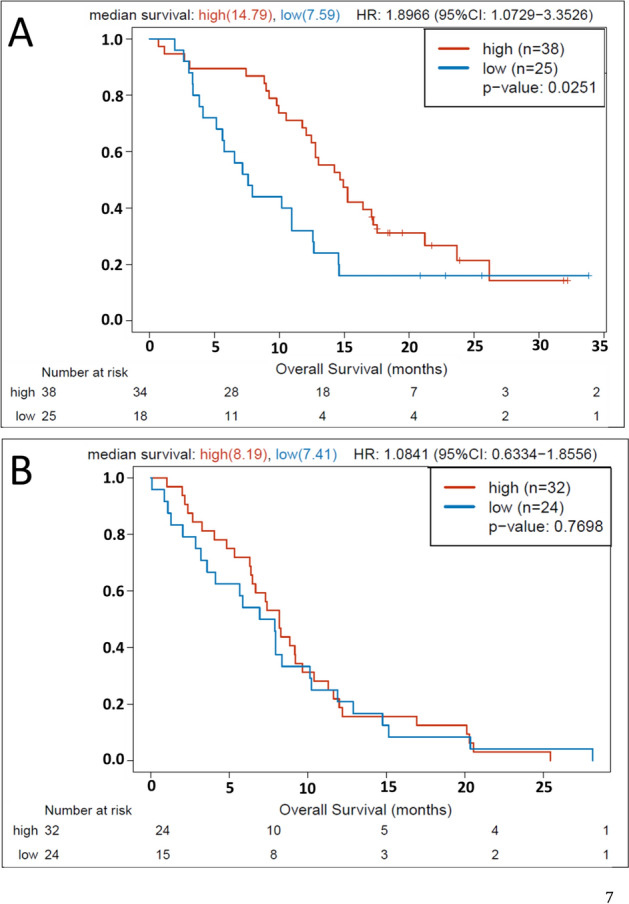
GATA6 IHC prediction of OS in advanced PDAC according to receipt of chemotherapy [**A**: in patients receiving first-line modified FOLFIRINOX (mFFX); **B**: in patients receiving gemcitabine–nabpaclitaxel (GnP)].

GATA6 IHC expression assessed with computer assistance was associated with overall survival (*P* = 0.04). When regrouped into low (0–2) and high (3–4) GATA6 IHC expression, low GATA6 IHC expression was associated with shorter overall survival [median OS was 6.2 months for patients with GATA6-low PDAC versus 11.5 months for patients with GATA6-high PDAC, hazard ratio 1.66 (95% CI 1.15–2.40), *P* = 0.007]. Median follow-up was 8.9 months. Figure 3 shows median OS according to chemotherapy regimens. In patients who received first-line modified FOLFIRINOX (mFFX; n = 63), median OS was 7.6 months in GATA6-low versus 14.8 months in GATA6-high subgroups [HR 1.90; 95% confidence interval (CI) 1.0729–3.3526; *P* = 0.025). In contrast, no significant difference was noted between subgroups in patients treated with gemcitabine–nabpaclitaxel [GnP], where median OS, was 7.41 months in GATA6-low versus 8.19 months in GATA6-high groups, respectively (HR 1.08; 95% CI 0.63–1.86; *P* = 0.77). An interaction analysis was also performed to evaluate whether there was a significant interaction between mFFX or GnP and the subgroups, and no statistically significant difference was noted to suggest one chemotherapy regimen for one particular subgroup.

### GATA6 IHC expression is associated with molecular subtypes and GATA6 RNA-seq levels

GATA6 IHC expression assessed with digital assistance was associated with modified Moffitt subtypes (*P* = 0.0003), Collisson subtypes (*P* = 0.004), and Bailey subtypes (*P* = 0.0001), with higher basal-like (Moffit), quasimesenchymal (Collisson), and squamous (Baily) subtype tumors in the GATA6-low group. GATA6 IHC expression assessed with digital assistance was also associated with GATA6 RNA-seq gene expression (*P* < 0.0001).

### Computer assistance improves GATA6 IHC evaluation

**Figure 4:**
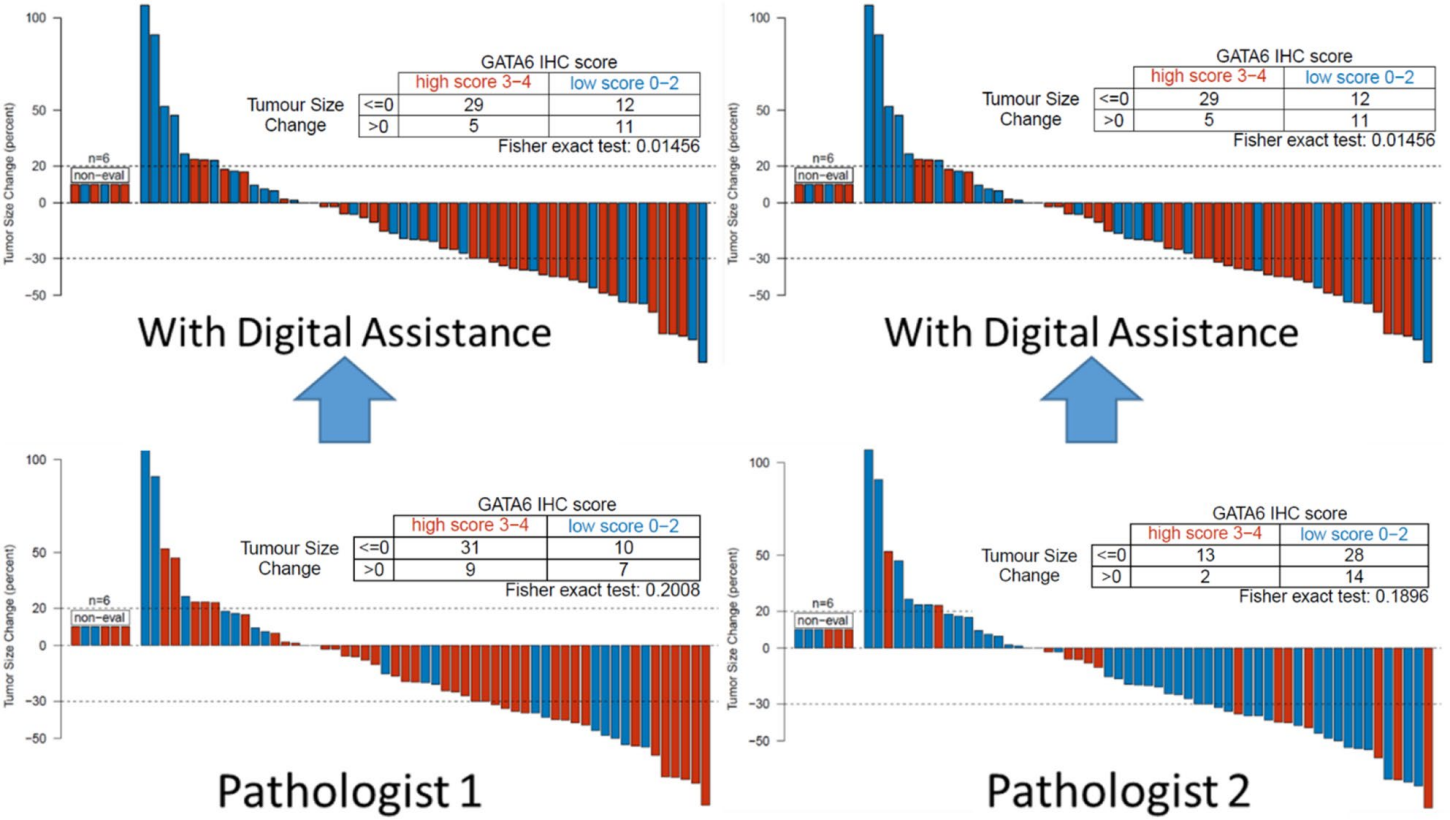
Digital assistance improves GATA6 IHC prediction of tumor size change in patients receiving first-line modified FOLFIRINOX (mFFX) chemotherapy. Waterfall plots demonstrating tumor size change according to low (0–2) and high (3–4) GATA6 IHC expression assessed with computer assistance (top panels) and without computer assistance (bottom panels).

**Figure 5:**
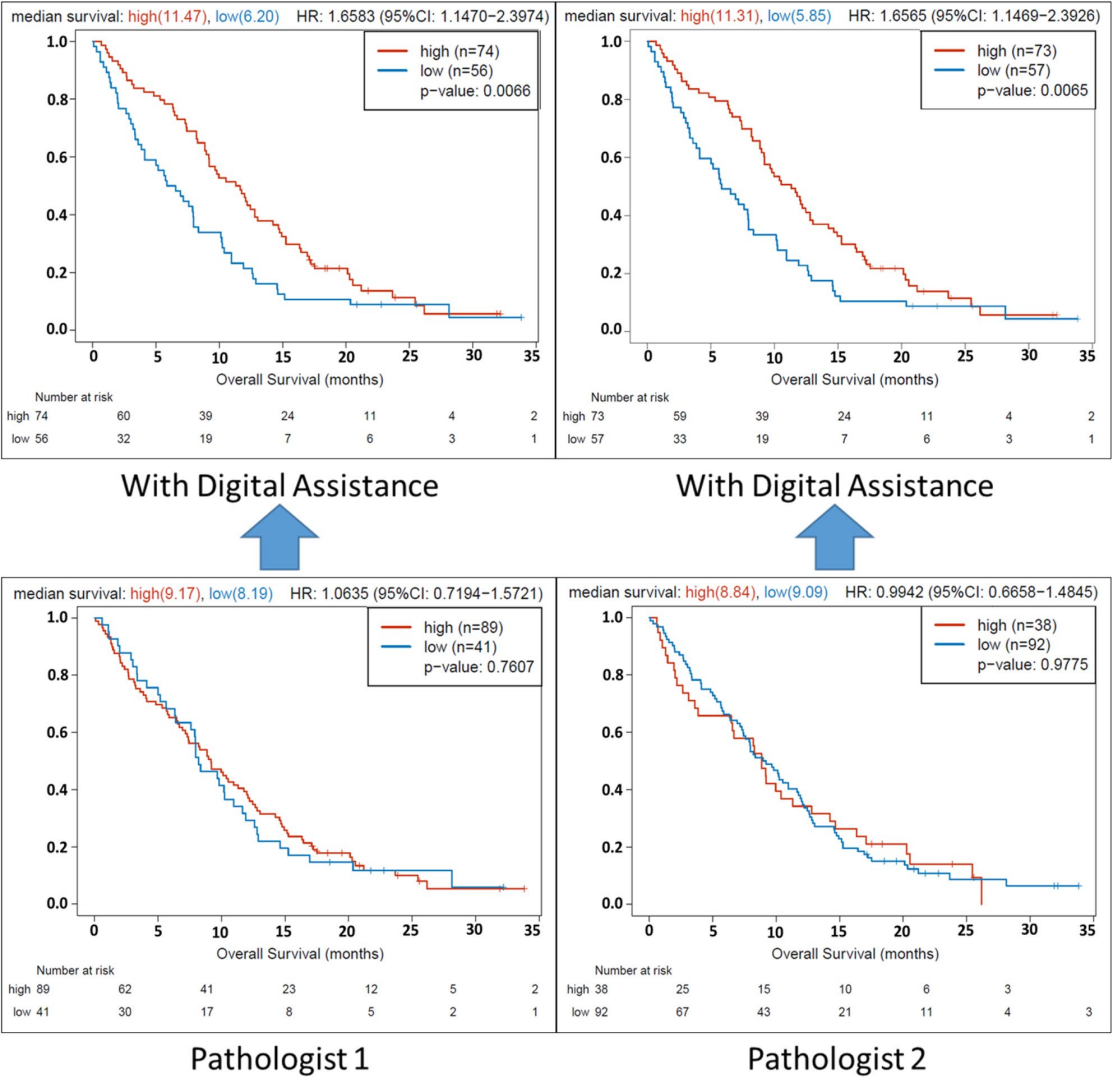
Digital assistance improves GATA6 IHC prediction of OS in advanced PDAC. Kaplan–Meier OS curves according to GATA6 IHC low and high expression assessed with computer assistance (top panels) and without computer assistance (bottom panels).

Computer assistance markedly improved interobserver concordance between pathologists (Cohen’s kappa scores of 0.95 vs. 0.32, with and without computer assistance respectively). Computer assistance also improved the association between GATA6 IHC expression and tumor size change in patients receiving first-line mFFX (*P* = 0.015 vs. *P* = 0.20 for pathologist 1; *P* = 0.015 vs. *P* = 0.19 for pathologist 2, with and without computer assistance respectively; Fig. 4). Furthermore, digital assistance improved GATA6 IHC prediction quality with regards to overall survival (*P* = 0.04 vs. *P* = 0.36 for pathologist 1; *P* = 0.04 vs. *P* = 0.97 for pathologist 2, with and without computer assistance respectively). When regrouped into low (0–2) and high (3–4) expression, digital assistance markedly improved the association between low GATA6 IHC expression and shorter overall OS (*P* = 0.007 vs. *P* = 0.76 for pathologist 1; *P* = 0.007 vs. *P* = 0.98 for pathologist 2, with and without computer assistance respectively; Fig. 5), particularly in patients receiving first-line mFFX (*P* = 0.0251 vs. *P* = 0.46 for pathologist 1; *P* = 0.0251 vs. *P* = 0.90 for pathologist 2, with and without computer assistance respectively). Digital assistance improved the association between GATA6 IHC expression and RNA-seq expression levels (*P* < 0.0001 vs. *P* = 0.05 for pathologist 1; *P* < 0.0001 vs. *P* = 0.04 for pathologist 2, with and without computer assistance respectively). Digital assistance also improved GATA6 IHC prediction quality with regards to modified Moffitt subtypes (*P* < 0.0001 vs. *P* = 0.11 for pathologist 1; *P* < 0.0001 vs. *P* = 0.04 for pathologist 2, with and without computer assistance respectively).

## Discussion

Combination chemotherapy, either modified FOLFIRINOX (mFFX) or gemcitabine–nabpaclitaxel (GnP), are used in the treatment of most patients with advanced PDAC, yet robust biomarkers of outcome are currently lacking to guide regimen selection^2–4^. Recently, our group demonstrated that patients with tumors of a modified “basal-like” phenotype are particularly resistant to mFFX and can be identified by low GATA6 expression by RNA sequencing or in situ hybridization^2^. However, the clinical applicability of these platforms remains limited by technical cost, accessibility, and reporting time. GATA6 IHC is therefore a more attractive option, because it is less costly, more widely available and can be readily integrated in the clinic by pathologists on routine FFPE tissue at the time of diagnosis. However, based on earlier experience^2^, we found accuracy to be lower for GATA6 IHC, possibly due to the subjective nature of visual assessment of staining intensity.

Recently, advances in digital pathology and image analysis have raised the possibility for computer assistance to improve manual assessment of IHC biomarkers^10–12^. Here, we show that GATA6 IHC expression assessed with computer assistance predicts overall survival in advanced PDAC. After an initial analysis of all five levels, we found that regrouping GATA6 IHC expression levels into low (0–2) and high (3–4) expression was a strong predictor of clinical outcome. Furthermore, we demonstrated that digital assistance, using a customized computer algorithm based on the open source software QuPath^15^, can improve interrater concordance and prediction of advanced PDAC clinical outcome using GATA6 IHC.

Although machine learning (ML)-based computer algorithms have recently been shown to improve pathologist performance on various diagnostic tasks, our results show that even non-ML digital image analysis tools have value that can be combined with human expertise to improve biomarker assessment^10–12^. Provided that validation studies are carried out according to consensus recommendations^16^, computer assistance offers exciting opportunities for pathologists to improve patient care. At this time, stand-alone computer algorithms are far from replacing the breadth and contextual knowledge of human experts, and this study demonstrates the potential benefits of a thoughtfully designed digital assistant to augment, rather than replace, pathologists for certain subjective diagnostic tasks such as evaluating IHC staining intensity, which may be prone to inter-observer variability.

In this study, pathologists independently assessed GATA6 IHC expression on FFPE tissue of advanced PDAC. While this is reflective of real-world practice, there are nevertheless limitations with this approach. In routine practice, pathologists may consult colleagues, which may improve assessment in borderline cases through consensus compared to individual performance in this study. Furthermore, although digital pathology is gaining more widespread adoption, this technology is not yet available in all pathology labs. Moreover, the digital assistant is not fully automated, as it relies on human expertise to distinguish tumor from non-tumor cells. Future ML-powered algorithms may automate tumor cell detection, which could further improve accuracy and precision of GATA6 IHC assessment. Since the digital assistant relied on pathologists to render the final score, we could not formally evaluate for false positive or false negative human interpretations due to digital overreliance. Future computer algorithms with more automation should consider the potential unintended effects of decision support tool bias on human performance. Finally, our study was limited to 130 advanced PDAC cases. Future prospective multi-institutional studies using larger datasets and pathologists with a broader range of backgrounds will ultimately be required to validate our results and determine whether these are generalizable to the general pathology community.

In this retrospective study, we confirm the prognostic utility of GATA6 IHC as a single important biomarker in advanced PDAC. Our findings provide further support to previous observations from Martinelli and colleagues showing that GATA6 loss in resected PDAC was associated with a basal-like phenotype in the ESPAC-3 trial, and shorter survival when treated with adjuvant 5-FU. Furthermore, Martinelli and colleagues also showed that GATA6-low cell lines derived from patient-derived xenografts were particularly resistant to 5-FU but not gemcitabine^17^. While the exact mechanism underlying the lack of response to 5-FU in GATA6-low tumors remains unclear, GATA6 has been recently shown to play an important role in regulating epithelial-mesenchymal transition and tumor dissemination in pancreatic cancer cells, and GATA6 loss appears to be associated with EGFR pathway activation in PDAC cells, suggesting perhaps a role for treatment response in patients^17^. Interestingly, in our study, we found that progression was higher in GATA6-low tumors compared to GATA6-high tumors in patients treated with mFFX but not in patients treated with gemcitabine regimens.

In summary, our study demonstrates that GATA6 IHC can be used as a single biomarker to predict clinical outcome in advanced PDAC, warranting further investigation in prospective clinical trials. We also show that digital assistance can markedly improve pathologist assessment of GATA6 IHC. These results provide the basis for an improved classification of PDAC and future biomarker design using computer-assisted pathology workflow.

## Abbreviations

PDAC: Pancreatic ductal adenocarcinoma
GnP: Gemcitabine–nabpaclitaxel
mFFX: Modified FOLFIRINOX
IHC: Immunohistochemistry
RNA-seq: RNA sequencing
ISH: In situ hybridization
FFPE: Formalin-fixed paraffin embedded
OS: Overall survival
ORR: Overall response rate
